# Hidden paths to endless forms most wonderful: Complexity of bacterial motility shapes diversification of latent phenotypes

**DOI:** 10.1186/s12862-020-01707-3

**Published:** 2020-11-04

**Authors:** Olaya Rendueles, Gregory J. Velicer

**Affiliations:** 1 Institute for Integrative Biology, ETH Zurich, Universitätstrasse 16, 8092 Zurich, Switzerland; 2 Microbial Evolutionary Genomics, Institut Pasteur, CNRS, UMR3525, 75015 Paris, France

**Keywords:** Dual motility, Indirect evolution, Pleiotropy, Social bacteria, MyxoEE-3

## Abstract

**Background:**

Evolution in one selective environment often latently generates phenotypic change that is manifested only later in different environments, but the complexity of behavior important to fitness in the original environment might influence the character of such latent-phenotype evolution. Using *Myxococcus xanthus,* a bacterium possessing two motility systems differing in effectiveness on hard vs. soft surfaces, we test (i) whether and how evolution while swarming on one surface—the selective surface—latently alters motility on the alternative surface type and (ii) whether patterns of such latent-phenotype evolution depend on the complexity of ancestral motility, specific ancestral motility genotypes and/or the selective surface of evolution. We analysze an experiment in which populations established from three ancestral genotypes—one with both motility systems intact and two others with one system debilitated—evolved while swarming across either hard or soft agar in six evolutionary treatments. We then compare motility-phenotype patterns across selective vs. alternative surface types.

**Results:**

Latent motility evolution was pervasive but varied in character as a function of the presence of one or two functional motility systems and, for some individual-treatment comparisons, the specific ancestral genotype and/or selective surface. Swarming rates on alternative vs. selective surfaces were positively correlated generally among populations with one functional motility system but not among those with two. This suggests that opportunities for pleiotropy and epistasis generated by increased genetic complexity underlying behavior can alter the character of latent-phenotype evolution. No tradeoff between motility performance across surface types was detected in the dual-system treatments, even after adaptation on a surface on which one motility system dominates strongly over the other in driving movement, but latent-phenotype evolution was instead idiosyncratic in these treatments. We further find that the magnitude of stochastic diversification at alternative-surface swarming among replicate populations greatly exceeded diversification of selective-surface swarming within some treatments and varied across treatments.

**Conclusion:**

Collectively, our results suggest that increases in the genetic and mechanistic complexity of behavior can increase the complexity of latent-phenotype evolution outcomes and illustrate that diversification manifested during evolution in one environment can be augmented greatly by diversification of latent phenotypes manifested later.

## Background

Understanding the causes of phenotypic evolution, including diversification, is a core motivation of evolutionary research [1–3]. This endeavor frequently focuses on adaptive phenotypic evolution in a focal selective context, but phenotypes also frequently first evolve non-adaptively [4–10]. Temporally, an initially non-adaptive phenotype might be manifested immediately when its causal genotype first evolves or might be latent until that genotype is exposed to a new environment. Here we use ‘latent-phenotype evolution’ (LPE) to refer to the initial evolution in one environment of the genetic basis of a focal phenotype that is only manifested later under different environmental conditions. While LPE per se is intrinsically non-adaptive, the allelic basis of LPE might originate adaptively (pleiotropy [6]), non-adaptively but driven by selection (hitchhiking [7, 8, 11]) or stochastically (genetic drift [9, 10]) and the focal latent phenotype might be beneficial, neutral or detrimental upon being manifested under different environmental conditions.

Phenotypes arising from LPE can be traits expressed by individual organisms (or genotypes), for example microbial genotypes arising in evolution experiments [9, 12, 13]. However, outcomes of organismal interactions can also be considered phenotypes—interaction phenotypes [14, 15]—that can also evolve latently. For instance, in allopatric speciation, reproductive incompatibility (an interaction phenotype) evolves latently during divergence of separate populations and is only revealed upon their secondary contact [16, 17]. While latent-phenotype evolution is clearly a significant contributor to overall biological diversification [6, 10, 12, 16–19], many questions regarding potential roles of evolutionary history (i.e. genotype [20]) and environment in determining the character of latent-phenotype evolution and diversification remain underexplored [21].

Pleiotropy from adaptive mutations is often invoked as a likely cause of LPE [22] and is a common feature of complex organisms [23, 24]. The potential for both pleiotropy and epistasis increase with the genetic and behavioral complexity of behavior, and both are important factors in the evolution of complexity [25–27]. Pleiotropy can have both positive [6] and negative [28, 29] fitness effects and antagonistic pleiotropy is frequently hypothesized to generate evolutionary tradeoffs between distinct traits affected by shared loci (e.g. between early- vs. late-life reproduction [30]). Many experimental-evolution studies have used various species of microbes to test for hypothesized evolutionary tradeoffs [31–37], including between growth rate and yield [38, 39], growth at different temperatures [40] or resource levels [41, 42] and age vs. reproductive rate [43]. However, few studies have tested for either tradeoffs or positively correlated LPE generated during adaptive evolution of actively motile bacteria [44–49]. Here, we do so with *Myxococcus xanthus,* a predatory soil-dwelling bacterium that swarms socially, cooperatively forms multicellular fruiting bodies upon starvation [50] and has become a model organism for microbial social evolution [51, 52].

Two distinct motility systems allow *M. xanthus* to effectively swarm and search for prey in heterogeneous soil environments [53, 54]. One system traditionally known as “S-motility” is driven by extension and retraction of type IV pili (TFP) [55, 56] and requires close proximity of neighboring cells to function effectively. The other system, traditionally known as “A-motility”, can drive movement by isolated cells. A-motility appears to be driven by an inter-membrane Agl-Glt motor complex that localizes at focal adhesion sites in contact with the substrate surface, but details of how motive force is generated remain to be resolved [57, 58]. These two motility systems contribute differently to swarming in distinct ecological contexts. Whereas movement on soft, wet surfaces (e.g. 0.5% nutrient agar) is driven primarily by Type IV pili (TFP) mediated S-motility, A- and S-motility make much more symmetric contributions to swarming across stiffer substrates such as 1.5% agar [53, 59–61]. Debilitation of the A-motility system alone (A−S+) has little or no effect on soft-agar swarming but substantially diminishes swarming on hard agar. Genotypes lacking S-motility while retaining A-motility (A+S−) exhibit only minimal swarming on soft agar compared to A+S+ while also showing reduced but nonetheless substantial swarming on hard agar [53, 59–61]. Dozens of genes affect swarming by each motility system and some affect both [62, 63].

In a previously reported evolution experiment [46–49, 61, 64] here named MyxoEE-3 (for ‘Myxobacteria Evolution Experiment 3’, see Methods), selection for increased fitness at the leading edge of actively growing and swarming *M. xanthus* colonies was imposed over hundreds of generations. Replicate populations starting from multiple ancestral motility genotypes adapted to a variety of different selective regimes [46–48]. MyxoEE-3 allows investigation of a variety of questions regarding evolution by bacteria undergoing largely continuous growth during active motility, including questions addressing modes of adaptation [47] and indirect evolution of latent phenotypes across a suite of traits [46, 48, 49].

Rendueles and Velicer (2017) examined modes of adaptation and phenotype evolution of MyxoEE-3 populations in the same selective environment in which each population evolved [47]. That study focused on six MyxoEE-3 treatments defined by the motility genotype of the founding ancestor—A+S+ (both systems intact), A−S+ (debilitated A-motility) or A+S− (debilitated S-motility)—and the agar-surface type on which they grew, swarmed and evolved—hard or soft nutrient CTT agar. Differential debilitation of each motility system (by deleting the *cglB* gene for the A−S+ ancestor [65] or the *pilA* gene for the A+S− ancestor [56]) allowed testing for both motility-genotype [20] and surface-type effects on the evolution of swarming rates and other potential modes of adaptation. Direct competition experiments showed that all MyxoEE-3 populations examined in [47] adapted to their selective environments, but only some did so by evolving faster intrinsic swarming on their MyxoEE-3 selective surface. Many populations did not evolve to swarm faster, with some even decreasing in swarming rate, implying that they adapted by alternative mechanisms, including potentially interference competition, increased intrinsic cell-division rate or facultative modulation of swarming rate in the presence of competitors [47].

Here we analyze the same six MyxoEE-3 treatments as examined in [47] to ask whether and how evolution while adapting to one surface type—the selective surface—latently shaped fitness and swarming phenotypes later revealed on the alternative surface to which each population did not adapt. In particular, we test among three hypothetical LPE outcomes for swarming-rate relationships on alternative vs. selective surfaces—positively correlated, negatively correlated or idiosyncratic LPE. By comparing populations derived from the three distinct ancestral motility genotypes (A+S+ , A−S+ , A+S−), we test for genotypic effects on LPE. Most broadly, by comparing LPE patterns across dual-system vs. single-system population categories, we test for LPE differences associated with different degrees of complexity in functional motility machinery. By comparing dual-motility-system populations across MyxoEE-3 environments, we test for an effect of surface type when surfaces differ with respect to the relative contributions of the two motility systems to movement. By comparing single-system populations across MyxoEE-3 environments, we test whether surface type matters for the character of LPE when only one motility system can be used during adaptation.

Since each ancestral motility system can alone drive at least some swarming on both surface types [53, 59–61], swarming-rate increases in the single-system MyxoEE-3 populations during adaptation to one surface might be expected to often carry over positively to the other surface and thereby generate positively correlated LPE. Nonetheless, the total gene sets contributing to swarming on hard vs. soft agar are likely to differ even for single-system genotypes, as are optimal expression patterns for genes implicated in swarming on both surfaces, such that LPE outcomes other than positive correlation among the single-system populations were plausible possibilities. In the A+S+ dual-motility populations, the functionality of both motility systems generates greater potential for evolutionary interactions between the two systems—and corresponding effects on the character of LPE—than in the populations lacking a gene essential to the operation of one system. Little is known about potential pleiotropic effects of the vast number of mutations that could occur among the many dozens of *M. xanthus* motility genes, making prediction of LPE outcomes for swarming rates seemingly even more difficult for dual-system than for single-system populations.

The design of MyxoEE-3 also allowed us to ask two questions regarding stochastic evolutionary diversification of latently evolved alternative-surface swarming rates. Within treatments, we ask whether the degree of stochastic diversification of alternative-surface swarming among replicate populations ever differs from, and possibly exceeds, stochastic diversification of selective-surface swarming. Across treatments, we ask whether the degree of stochastic diversification of latently evolved alternative-surface swarming can be contingent on the starting genotype or physical environment of adaptive evolution.

## Results

### Alternative-surface fitness increased, but often less than selective-surface fitness

Surfaces differing in features such as terrain, matter state or moisture content can differ radically in what mechanisms optimize organismal locomotion across them and hence in the adaptive landscapes [66] they impose on motile organisms [59]. We first test whether the adaptive landscapes of each ancestral genotype differ across surface types by asking whether selective-surface fitness gains tend to carry over to the alternative surface or not. If fitness patterns of the evolved replicate populations of a given treatment differ significantly between their selective and alternative surfaces, this implies that the adaptive landscape of their shared experimental ancestor differs between the two surface types.

We used a coarse measure of fitness to test whether fitness gains achieved by evolved populations on their selective surface might carry over equally to the alternative surface. Each evolved population was mixed at a 1:1 initial ratio with a kanamycin-resistant variant of its ancestor and the resulting mixed colonies were allowed to swarm outward for seven days on both the selective and alternative agar type for each population, after which the presence or absence of the ancestor at multiple points around the swarm perimeter was scored. In control assays in which the marked variants of the experimental ancestors were competed with their unmarked parents, the marked ancestors were present in either 100% or nearly 100% of all post-swarming edge-population samples (Additional file 1, Table S2), indicating that the marked ancestors do not have major, if any, fitness defects during active swarming. In the competitions with evolved populations, consistent reductions in the proportion of edge samples containing the ancestor relative to the controls reflect increased fitness by the evolved populations.

We reported previously that for competitions performed on populations’ MyxoEE-3 selective surfaces, 78% (43/55) of examined populations completely excluded the marked ancestor from post-competition perimeter samples in all samples in all replicates [47]. Here we find that a significantly lower fraction of evolved populations completely excluded their ancestor from the swarm edge on their alternative surface (23/55 populations, 41%, one-tailed Fisher’s exact test, *p* < 0.001) (Additional file 1, Fig. S1 and Table S2), indicating that latent fitness gains carrying over to the alternative surfaces clearly tended to be lower than direct fitness gains on the selective surfaces. In turn, this shows that the ancestral genotypes have different fitness landscapes for hard and soft CTT agar.

**Fig. 1:**
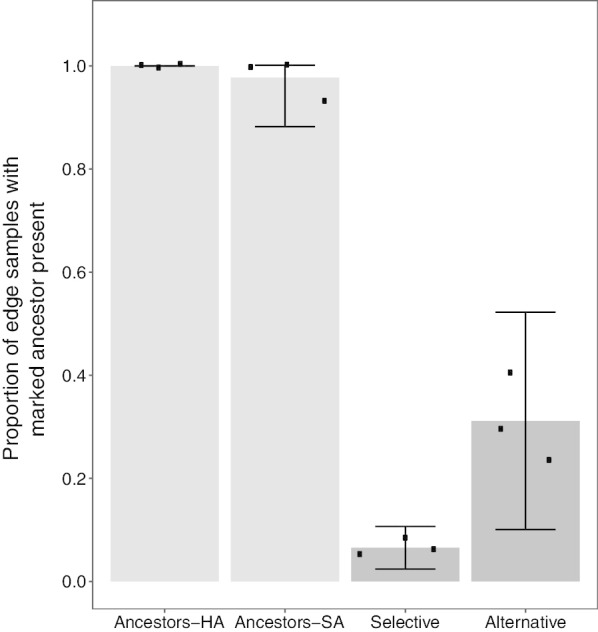
Alternative-surface fitness increased less than selective-surface fitness. Marked ancestors were present in nearly 100% of all post-competition swarm-edge population samples after control competitions with unmarked ancestors on hard and soft agar. After competition with evolved populations, the marked ancestors were present in only ~ 7% of samples for competitions carried out on their MyxoEE-3 selective surface but were present in ~ 31% of samples after competitions on the populations’ alternative surfaces. Data points represent within-replicate ancestor-presence proportion averages for each category, gray bars show cross-replicate means and error bars show 95% confidence intervals. *HA* hard agar, *SA* soft agar

For each evolved population, we additionally calculated the mean proportion of edge samples, across replicate assays, in which the marked ancestor was detected and then compared outcomes between selective and alternative surfaces. First averaging across all populations, irrespective of treatment, yields the same conclusion as the previous analysis, namely that alternative-surface fitness was lower overall than selective-surface fitness. Specifically, the average ancestor-presence frequency among population samples per competition plate was significantly higher on alternative surfaces (0.315) than on selective surfaces (0.065) across all 55 populations collectively (*p* = 0.01, one-tailed paired *t-*test, Fig. 1). However, the degree of fitness difference between surface types differed between some treatments when the six treatments were considered individually (Additional file 1, Fig. S1).

### Stochastic diversification of colony phenotypes

**Fig. 2:**
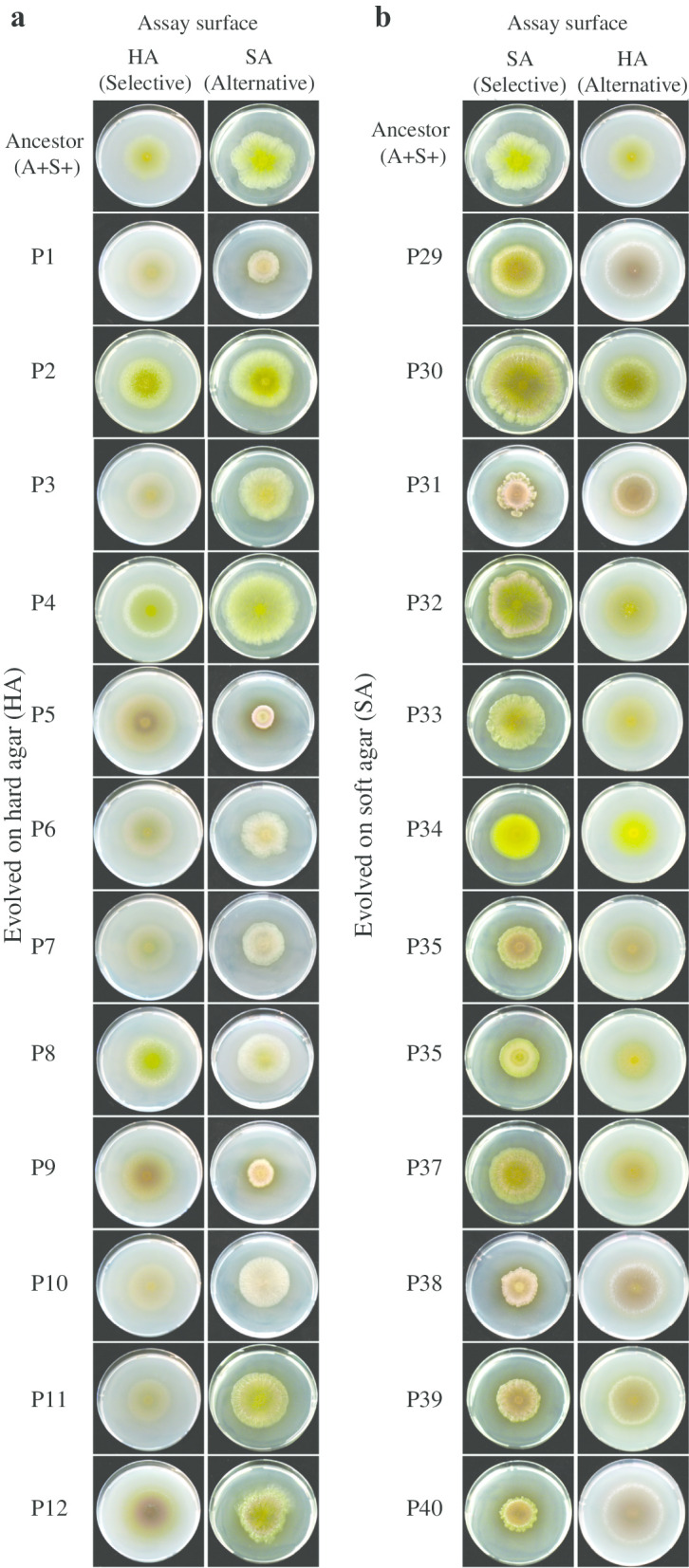
Colony-phenotype diversification of A+S+ populations on both surface types. Colony phenotypes of **a** twelve populations evolved on hard agar (A+S+ HA), **b** twelve populations evolved on soft agar (A+S+ SA) and their ancestors (in both **a** and **b**) on both their selective and alternative surface types

Evolved populations were allowed to swarm on their alternative-surface in the absence of the ancestor. These assays revealed that swarm color and overall morphology clearly diversified stochastically among replicate populations within most treatments (Fig. 2, Additional file 1, Figs. S2 and S3). We did not visually detect obvious major differences in diversification patterns expressed on selective vs. alternative surfaces with respect to phenotypic ranges or trends, except for A+S− populations. These populations evolved on soft agar (P49–P56) diversified in their soft-agar colony morphologies to a greater degree than did the A+S− populations evolved on hard agar (P21–P28) due to populations P55 and P56 (‘E7’ and ‘E8’ in [61]), which evolved new colony-level morphologies along with increased swarming rates on soft agar. Because replicate populations adapted to the same selective environment, the evolution of heritable phenotypic variation among replicate populations in any given treatment can only be explained by stochastic variation in the identity and or/sequence of mutations occurring across populations. This highlights the major role of chance in shaping phenotypic evolution [21, 47, 67, 68].

### Alternative-surface swarming did not increase at the treatment level

In the same assays, alternative-surface swarming rates varied among evolved populations within each treatment (Additional file 1, Fig. S4A, S4B; Table S3) and also in patterns at the treatment level. Treatments varied in the proportion of populations that exhibited significant change on the alternative surface, ranging from 12.5% (A+S− HA) to 71% (A−S+ SA) and in whether any populations evolved significantly reduced alternative-surface swarming, as did the A+S+ SA and A−S+ SA treatments. As previously reported, average motility rates among replicate evolved populations increased significantly on their selective surface in three treatments: A−S+ HA and A+S− HA and A+S+ SA [47]. However, none of these treatment-level increases in swarming rate on the selective surface translated into significant increases on the alternative surface (Additional file 1, Fig. S4C and Table S4).

### Dual-system populations collectively exhibit idiosyncratic LPE

**Fig. 3:**
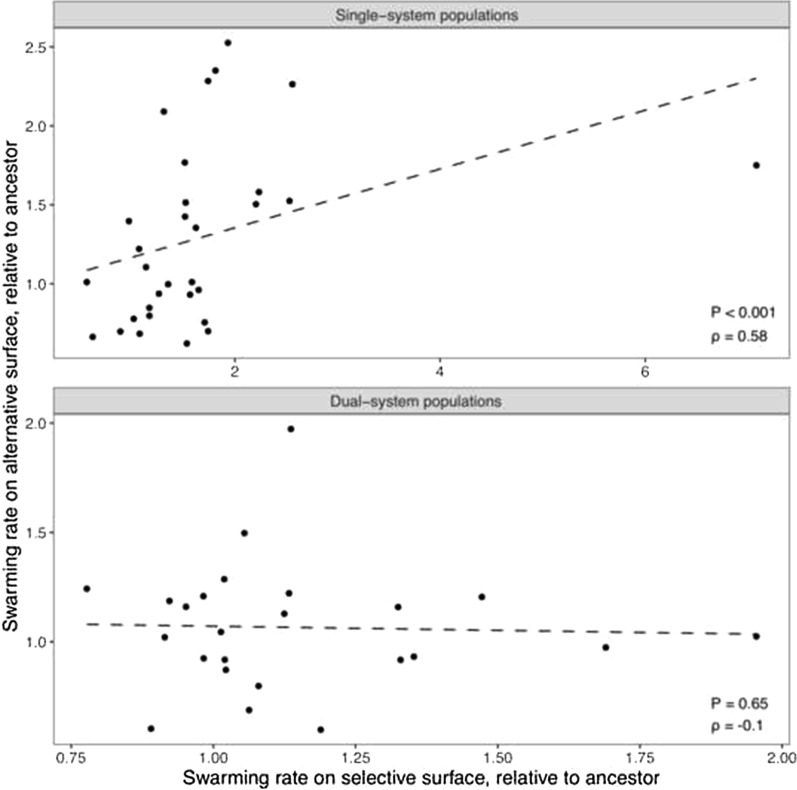
Alternative-surface swarming correlates positively with selective-surface swarming among single-motility-system populations but not dual-motility populations. Plotted values represent means of evolved/ancestral swarming-rate ratios across at least three replicate experiments. Dashed lines represent the linear model fit. *p* and rho values correspond to Spearman correlation tests. The positive correlation in single-system populations remains significant when the outlier is removed (*P* = 0.003 rho = 0.53, see Additional file 1, Fig. S6)

Swarming rates of *M. xanthus* natural isolates from a cm-scale population were previously found to correlate positively across hard vs. soft agar [69]. Under the assumption that both A- and S-motility are functional in most natural isolates, this correlation suggested that evolutionary change in swarming rates on selective vs. alternative surfaces might correlate positively for the A+S+ populations in our experiments. However, latent swarming-rate evolution in the A+S+ treatments of MyxoEE-3 is found to be idiosyncratic in character. No correlation, positive or negative, in swarming-rate evolution across selective vs. alternative surface types is observed within the A+S+ treatments, whether considered collectively or individually (Fig. 3 and Additional file 1, Fig. S5, respectively; Additional file 1, Table S5). Nor was there a significant tendency toward either positive or negative changes on the alternative surface, with 14 positive estimates and ten negative (Additional file 1, Table S3). Among eight populations that increased most strongly in swarming rate on their selective surface (P4, P5, P8, P29, P30, P32, P34, and P38; Additional file 1, Fig. S4, Table S3), only three (P4, P8 and P38) appear to have correspondingly increased on the alternative surface. Moreover, four populations that increased swarming rate strongly on the alternative surface did not do so on their selective surface (P2, P10, P33 and P40).

The A+S+ treatments allow us to consider evolutionary relationships between the two motility systems of *M. xanthus* and swarming patterns across the surface types*.* As noted above, swarming on soft agar by the ancestral A+S+ genotype is driven primarily by TFP-mediated S-motility, whereas both motility systems contribute substantially to movement on hard agar (Additional file 1, Fig. S4) [53, 61]. Among the A+ H+ HA populations, several underwent substantial indirect evolution of soft-agar swarming rate, suggesting that evolution in this treatment was not driven solely by genes specific to A-motility but also involved genes contributing to S-motility in some populations.

Among the A+S+ SA populations, three (P33, P36 and P40) showed substantial increases in their hard-agar swarming rate. Others populations showed no such positive LPE despite having increased on soft agar (e.g. P30, P32 and P34) (Additional file 1, Figs. S4, S5; Table S3). Because swarming on soft agar is driven almost exclusively by S-motility in the A+S+ ancestors, these outcomes suggest that some populations underwent evolution in S-motility loci that contribute solely to soft-agar swarming (e.g. P30, P32 and P34) whereas others evolved at S-motility loci that affect swarming on both surfaces. It is also possible that A-motility loci evolved to affect soft-agar swarming despite having no such effect in their ancestral state [61].

### Single-system treatments collectively exhibit positively correlated LPE but vary across individual treatments

Unlike the A+S+ populations, LPE was strongly positively correlated across surface types among the populations descended from single-system ancestral genotypes (A−S+ and A+S−, irrespective of surface type), (Fig. 3 and Additional file 1, Table S5). Single-system populations evolved on soft agar contributed more strongly to the correlation than populations evolved on hard agar (Additional file 1, Table S5). Compared with the absence of such a correlation among the A+S+ populations, this outcome suggests that the presence of multiple operational motility systems complexifies LPE outcomes across replicate populations, on average, compared to the presence of only one system.

Among the four single-system treatments considered individually (Additional file 1, Fig. S5), the A−S+ SA populations lacking the *cglB* gene generated the most strongly correlated LPE. Comparing this outcome to the lack of correlated evolution in the A+S+ SA treatment implies that the presence of *cglB* prevented correlated evolution across surface types even though *cglB* has no detectable effect on soft-agar motility in the ancestral genomic background (Additional file 1, Fig. S4) [47]. This result indicates that a genic/allelic state that exerts little phenotypic effect in a focal selective environment can nonetheless greatly shape the character of LPE.

Despite the overall correlation of LPE across all single-system treatments together and each single-system genotype (irrespective of selective surface) (Additional file 1, Table S5), the A−S+ HA treatment considered alone did not exhibit even a suggested correlation. The contrast of this outcome with that of the A−S+ SA treatment shows that selective environment can sometimes shape the character of LPE for a given ancestral genotype.

### Latent swarming-rate increases by individual populations

Novel traits or increased trait function can evolve due to selection for such gains per se [61, 70, 71] but can also evolve indirectly without selection [46, 48, 72, 73]. A substantial minority of MyxoEE-3 populations indirectly evolved faster swarming on their alternative surface type (Fig. 3, Additional file 1, Fig. S5; Table S3), but the mechanistic implications of such gains differ across treatments. As noted above, S-motility alone in the ancestral state (A−S+) drives reduced yet nonetheless substantial swarming on hard agar relative to the A+S+ ancestor and also drives swarming on soft agar that is indistinguishable from A+S+ (Additional file 1, Fig. S4 and [61]). The functionality of S-motility on both surface types suggests that selective-surface swarming increases by A−S+ populations might often carry over to enhance swarming on the alternative surface as well. This was the case for several populations (e.g. P13, P18, P19, P42, P43 and P45; Additional file 1, Table S3), at least under the assumption that the same mutations are generally responsible for increased swarming on both surfaces.

In contrast to the residual ability of A−S+ genotypes to swarm on hard agar, A-motility alone (in A+S− genotypes) drives only little colony expansion on soft agar (Additional file 1, Fig. S4, [53, 61]). A previous study and our new assays with eight A+S− populations (P49–P56 here, ‘E1–E8’ in ref [61]) have shown that A+S− populations can evolve novel forms of effective soft-agar swarming while under selection on soft agar ([61], Additional file 1, Fig. S3). We find that soft-agar motility by A+S− genotypes can also increase indirectly under selection on hard agar. Specifically, the soft-agar swarming of P24 increased more than two-fold in association with faster swarming on hard agar (Additional file 1, Fig. S5; Table S3). However, most A+S− HA populations did not evolve significantly in their soft-agar swarming rate (Additional file 1, Fig. S4; Table S3), pointing to diversity in the pleiotropic character of mutations that accumulated during adaptation of A+S− populations to hard agar.

Latent swarming-rate increases on the alternative surface sometimes exceeded increases on the selective surface (e.g. P4, P18, P24, P43 and P51; Additional file 1, Fig. S5; Table S3), highlighting the potential for latent side-effects of adaptive evolution to be of greater phenotypic magnitude than directly adaptive effects. Most strikingly, at least one population (P40) evolved increased swarming on its alternative surface (faster swarming on hard agar) despite an apparent decrease in swarming rate on its selective surface (Additional file 1, Fig. S5; Table S3).

### Stochastic diversification of latent phenotypes varies quantitatively across evolutionary treatments

Divergence between replicate experimental populations adapting independently to identical conditions is explained by stochastically differential mutational input [21]. Using a parameter that quantifies diversification independently of the mean degree of trait change (*I*_*x*_*,* [47, 74]), we previously demonstrated that the MyxoEE-3 populations examined here exhibited high levels of within-treatment diversification with respect to swarming rate on their selective surface, as compared to diversification among swarming *Pseudomonas aeruginosa* populations in another study [44, 47]. Using the same parameter, here we ask whether stochastic diversification of alternative-surface swarming rates among replicate populations within treatments ever differs in magnitude, and not just phenotypic character (Fig. 2), from that of selective-surface swarming rates. Across treatments, we ask whether relative patterns of alternative- vs. selective-surface degrees of diversification tend to be similar or rather differ by ancestral genotype and/or MyxoEE-3 selective surface.

**Fig. 4:**
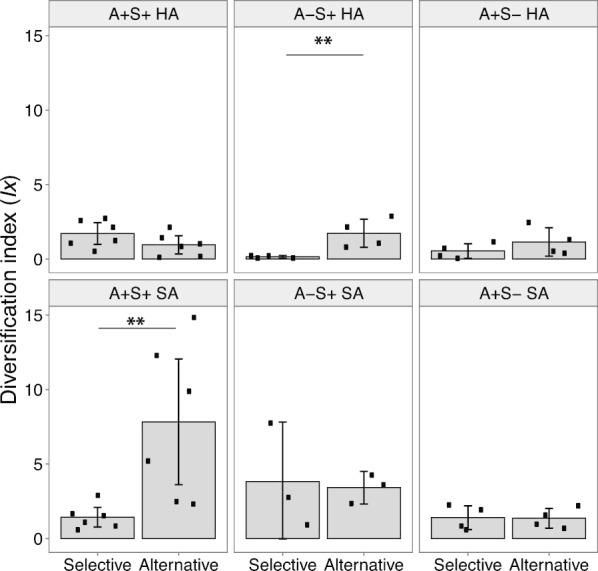
Stochastic diversification of evolved replicate populations can be greater on an alternative surface than on their selective surface and varies across treatments. Within-treatment diversification index (*I*_*x*_) for swarming rates. *I*_*x*_ values here represent the average divergence among multiple independently evolved pairs of strains that each descended from the same ancestor and evolved in the same environment. Each individual point indicates the diversification index of a unique pair. **p < 0.01 for significant difference in a student *t*-test. Error bars show 95% confidence intervals

Degrees of latent stochastic diversification within treatments manifested on alternative surfaces varied greatly across evolutionary treatments, both relative to diversification on the selective surface and comparing directly across treatments (Fig. 4). We find that the magnitude of latent diversification manifested in a new environment can exceed diversification observable in the original selective context, as alternative-surface diversification in the A−S+ HA and A+S+ SA treatments was significantly higher than selective-surface diversification (Fig. 4). Moreover, the variation in alternative-surface diversification observed across treatments (both in absolute terms and relative to selective environments) indicates that the genomic-environmental context in which adaptive evolution occurs can shape the magnitude of latent stochastic diversification.

## Discussion and conclusion

Several types of LPE have previously been demonstrated with MyxoEE-3 populations. For example, colony-merger incompatibility, a form of kin discrimination, readily evolved among within-treatment replicate populations as a byproduct of differential evolution during adaptation to a common environment [48]. Nair and Velicer showed that facultative social exploitation and other interaction-specific fitness inequalities during multicellular development evolved among MyxoEE-3 populations despite the absence of experimental selection on development [46]. Focusing on a single A+S− SA population (here P56), Zee et al*.* demonstrated that mutations accumulated during P56 evolution on soft agar had large indirect effects on several behaviors in different environments, including swarming on hard agar, predation and development during starvation [49].

Here we used six MyxoEE-3 treatments to examine how colony phenotypes of motile microbes latently evolve and diversify as a function of their starting genotype and selective surface. Selection for competitiveness at the leading edge of swarming *M. xanthus* colonies during evolution on one surface often had major effects on later swarming on an alternative surface. However, the character of such LPE differed as a function of multiple factors—the number of functional motility systems operating during MyxoEE-3 evolution, ancestral genotype, and selective surface (Fig. 3, Additional file 1, Fig. S5).

The most basic difference between the genotypic starting points of MyxoEE-3 treatments examined here was the presence of two functional motility systems or only one. The dually motile A+S+ treatments had greater potential for evolutionary interactions between the A and S systems because all genes necessary for effective operation of both systems were intact. Limited prior knowledge regarding such interactions in *M. xanthus* made all three major hypothetical outcomes of LPE correlation analyses with the A+S+ treatments—positive correlation, negative correlation or no correlation—all plausible a priori*.*

Positive LPE correlations among the A+S+ populations were plausible in light of a prior study that found a positive correlation between hard- vs. soft-agar swarming rates among *M. xanthus* natural isolates, which might be generally expected to have both motility systems intact [69]. Such a positive correlation might occur if many states of motility-related gene expression have similar effects on population-level swarming across the two surfaces. Negative LPE correlations were plausible in light of potential for energetic or mechanical tradeoffs between swarming on the two surfaces. For example, in a previous study with *P. aeruginosa*, experimental selection of dually motile populations for increased motility on hard (1.2%) agar or soft (0.3%) agar, driven predominantly by TFP and flagellar motility, respectively, tended to reduce motility on the alternative surface [44]. As a third possibility, lack of correlation due to idiosyncratic LPE was plausible because many motility-related genes might have been targets of selection [62, 63] and different adaptive mutations across those genes may often differ in their quantitative or qualitative effects on alternative-surface swarming. Finally, if the gene networks underlying swarming on the two surfaces were highly modular (i.e. share few loci) [75, 76], little or no indirect motility-rate evolution might have occurred. Our collective results with the A+S+ populations most strongly support the idiosyncracy hypothesis, as many populations appear to show evolutionarily change in their alternative-surface swarming rates (Additional file 1, Fig. S5), but not even a slight significant correlation between swarming rates on selective vs. alternative surfaces was detected (Fig. 3b).

In contrast, the genetically and mechanistically simpler category of single-system treatments collectively showed strongly positively correlated LPE (Fig. 3a). This different outcome suggests that differences in genomic complexity underlying behaviors such as motility can determine the character of LPE. The greater motility complexity of the A+S+ treatments appears to have allowed a greater range of LPE outcomes than were available to the single-system treatments, thus resulting in the absence of positively correlated LPE. This outcome illustrates that manipulating the complexity of genome space associated with traits of interest combined with experimental evolution and comparison of trait change in selective vs. alternative contexts offers much opportunity for investigating relationships between complexity and the character of LPE.

Because the A+S+ ancestors of MyxoEE-3 swarm faster than the single-system mutants on either one or both surface types examined here (Additional file 1, Fig. S4A, B), the A+S+ treatments would be predicted to have undergone more generations of evolution than the mutant populations in the absence of swarming-rate evolution [47]. In theory, such a difference in the degree of evolution undergone by the different categories might have contributed to the contrasting LPE outcomes shown in Fig. 3. In this scenario, the character of A+S+ LPE would have had to change over time, with early evolution of a positive LPE correlation later changing to uncorrelated LPE. However, results from genome sequencing of evolved clones from each population are not consistent with this hypothesis. As reported previously [47], the genome of one clone from each of the MyxoEE-3 populations examined here was sequenced and compared to its ancestor. Excluding a mutator clone from P29 [47], we find no significant difference in the average number of mutations present among A+S+ clones (13.2 mutations/clone, *n* = 23) vs. single-system mutant clones (14.3 mutations per clone, *n* = 31) (Additional file 1, Fig. S7). Because the difference in qualitative correlation outcomes reported between Fig. 3a and b is retained when the mutator P29 is excluded (Spearman’s rho = − 0.15 for the A+S+ dual-motility correlation, *p* = 0.48), this difference is not explained by different degrees of genomic evolution. The lack of difference in genomic evolution between the dual- vs. single-system categories may be partially explained by the outcome that the mutant populations tended to evolve greater proportional increases in selective-surface swarming rates during MyxoEE-3 (Additional file 1, Fig. S4C), thereby reducing differences in generation numbers predicted from ancestral swarming rates.

Comparing LPE outcomes across individual treatments show that differences of the specific genotypic starting point and ecological context of evolution can shape the character of LPE. For example, the A−S+ SA treatment exhibited positively correlated LPE for swarming rates. However, other treatments that differed only in ancestral genotype (e.g. A+S+ SA) or only in selective environment (A−S+ HA) clearly did not (Additional file 1, Fig. S5). Because correlated evolution (whether positive or negative) might occur when starting from one genotype but not from another, or, for the same starting genotype, might occur during adaptation in one environment but not another, experimental tests for LPE, including tests of tradeoff hypotheses [41, 42, 77], should be designed and interpreted in light of such potential for historical and environmental contingencies [41, 42, 77].

Within all MyxoEE-3 treatments, replicate populations diversified latently for alternative-surface swarming distinctly from selective-surface diversification, whether in colony morphology (Fig. 2, Additional file 1, Figs. S2 and S3) or swarming rates (Additional file 1, Fig. S5). These outcomes illustrate that much of the total phenotypic diversity that evolves stochastically among replicate experimental populations in a given environment [21] remains latent until populations are exposed to new conditions. Indeed, latent-phenotype diversification can in some treatments greatly exceed diversification manifested immediately in the selective environment (A−S+ HA and A+S+ SA, Fig. 4). Such patterns suggest that the replicate populations were evolving toward distinct fitness peaks in an adaptive landscape, beyond any such suggestion from phenotypic variation among populations observed in the original selective environment [67].

However, the magnitude of latent stochastic diversification can depend strongly on the presence or absence of a single gene (e.g. compare A+S+ SA vs. A+S− SA, Fig. 4) or on a simple difference in surface viscosity during adaptive evolution (compare A+S+ HA vs. A+S+ SA, Fig. 4). It has been previously recognized that distinct adaptive landscapes imposed by different selection regimes allow different degrees of stochastic diversification among replicate experimental populations in their selective environments [47, 67]. Our results show that distinct selective environments can also determine the degree of stochastic latent-phenotype diversification across replicate populations.

In most experimental evolution studies to date, it has not been clear what proportion of mutations that increased to high frequency did so because they were beneficial. A recent study with yeast suggests that the fraction of mutations that evolve by hitchhiking in asexual populations may often be high [11]. Thus, some LPE documented here might be caused by non-adaptive mutations that hitchhiked to high frequency. However, because all MyxoEE-3 populations exhibited strong adaptive gains on their selective surface [47], it is likely that much of the LPE documented here is caused by pleiotropy from adaptive mutations, a common feature of complex and social organisms [23, 24, 78, 79]. The relative contributions of adaptive vs. non-adaptive evolution to LPE in evolution experiments could be ascertained by identifying mutations underlying latent phenotypes and testing whether they were beneficial upon appearance.

## Methods

*Nomenclature* To facilitate reference to the broader evolution experiment of which the six treatments examined here were a part, we name the overall experiment ‘MyxoEE-3’, with ‘MyxoEE’ meaning ‘Myxobacteria Evolution Experiment*’* and ‘3’ indicating the temporal rank position of the first publication from this experiment [61] relative to the first publications from other evolution experiments using myxobacteria. The shared features of all MyxoEE-3 treatments have been described previously [46–48] and a list of distinct treatments can be found in Additional file 1, Table S3 of [46]. Other MyxoEEs from which studies have already been published are hereby correspondingly named MyxoEE-1 [80], MyxoEE-2 [81, 82], MyxoEE-4 [83], MyxoEE-5 [84] and MyxoEE-6 [85].

*Ancestral genotypes* In each of the six MyxoEE-3 treatments examined here, evolving populations were established from ancestral clones with one of three motility genotypes, with each ancestral motility genotype represented by both a rifampicin-sensitive strain and a rifampicin-resistant (rif^R^) mutant of the sensitive strain: (i) “wild type” (A+S+) strains GJV1 (itself derived from strain DK1622) [86] and its rif^R^ mutant GJV2, in which both motility systems are functional, (ii) A−S+ strains GJV3 and GJV5 (rif^R^), defective at A-motility due to deletion of *cglB*, and (iii) A+S− strains GJV4 and GJV6 (rif^R^), defective at S-motility due to deletion of *pilA* [59]*.* Each evolutionary lineage was established with an independently isolated sub-clone of the six above-mentioned ancestral genotypes [47, 48].

*Experimental evolution* Experimental evolution was carried out on the surface of CTT agar plates (8 mM MgSO_4_, 10 mM Tris pH 8.0, 10 g/L casitone, 1 mM KPO_4_) in two environments that differed only in agar concentration—0.5% (soft, aka “SA”) or 1.5% (hard, aka “HA”). The six evolutionary treatments defined by one of the three ancestral motility genotypes and one of the two evolutionary surface types are summarized in Additional file 1, Table S1. Either eight or twelve replicate populations per treatment (Additional file 1, Table S1) were grown at 32 °C, 90% rH, as follows: ten-µl aliquots containing ~ 5 × 10^8^ M*. xanthus* cells were placed in the center of a Petri-dish and allowed to grow and swarm for two weeks. At two-week intervals, an agar fragment (~ 3 × 5 mm) was cut out from the swarm perimeter furthest from the center (or from a random perimeter point for circular swarms) of each plate and placed on the center of a new plate on which the population was allowed to grow and swarm again for two weeks. This was repeated for 40 cycles, except for P14, which is examined after 36 cycles due to a contamination event (see Additional file 1, Table S1).

*Growth conditions* Prior to all post-evolution experiments, ancestral and evolved populations were inoculated from frozen stocks and grown on CTT hard-agar plates at 32 °C, 90% rH. Population samples were transferred into 8 mL CTT liquid and allowed to grow overnight at 32 °C with shaking (300 rpm) until mid-exponential phase (OD_600nm_ ~ 0.5). Cell densities were estimated with a TECAN Genios™ plate reader prior to initiating swarming-rate or competition assays. All plate cultures in all assays were incubated at 32 °C, 90% rH. HA and SA assays were performed simultaneously in each replicate and were inoculated from the same set of liquid cultures.

*Swarming-rate assays *The day before swarming assays were initiated, agar plates were poured (20 mL in 9-cm-diameter Petri dishes) and allowed to solidify uncovered for 15–20 min in a sterile laminar-flow hood before being covered and stored overnight at room temperature. To initiate the assays, liquid cultures were centrifuged and resuspended with CTT liquid to ~ 5 × 10^9^ cells/mL. Ten microliters of each resuspended culture were then placed at the center of an agar plate and incubated for seven days. Swarm perimeters were marked after one and four days of incubation and the distance swarmed between those time points for each replicate was estimated as the average distance along four perpendicular vectors with a random orientation. Pictures were taken after seven days.

*Competition experiments* As reported previously, *M. xanthus* cells of some strains, including some MyxoEE-3 evolved populations, cohere during growth on agar plates to a degree not readily overcome by experimental disaggregation methods, thus precluding the use of traditional techniques such as dilution plating and CFU counting [47]. We therefore assessed the fitness of evolved populations relative to their respective ancestors by scoring the presence or absence of kanamycin-resistant (kan^R^) variant of the respective ancestor at the leading edge of colonies [47]. To initiate the competition experiments, 10 µl of a kan^R^ ancestor:evolved mixed culture (1:1 initial ratio) were spotted onto the center of plates prepared as described above. Control assays with kanamycin-resistant and sensitive ancestral variants mixed at a 1:1 ratio were performed simultaneously. To test whether marked ancestors were present at the leading edge of swarming populations, samples from five locations evenly distributed around each swarm perimeter were harvested with a sterile tip and transferred onto CTT hard-agar plates with and without kanamycin (40 µg/mL). Results in Additional file 1, Table S2 reflect growth on antibiotic plates scored seven days after transfer.

All experiments were performed in three temporally separate replicate blocks.

## Supplementary information

**Additional file 1: Figure S1.** Alternative-surface fitness patterns across treatments: per-population means. The leading edges of swarming colonies initially mixed as 1:1 evolved:marked-ancestor were sampled for the presence/absence of the marked ancestor (five samples per colony). The mean ancestor-presence proportions for each evolved population across three replicate assays are shown (data points). Gray bars correspond to the within-treatment average of the per-population means. Data for the selective-surface assays was originally published in [47]. * *p* < 0.05, ***p < 0.001; Student’s *t-*test for proportion differences between selective vs. alternative surfaces. Error bars show 95% confidence intervals. **Figure S2.** Colony-phenotype diversification of A−S+ populations on both surface types. Colony phenotypes of (**a**) eight populations evolved on hard agar (HA), (**b**) seven populations evolved on soft agar (SA) and their ancestors (in both **a** and **b**) on both their selective and alternative surface types. **Figure S3.** Colony-phenotype diversification of A+S- populations on both surface types. Colony phenotypes of (**a**) eight populations evolved on hard agar (HA), (**b**) eight populations evolved on soft agar (SA) and their ancestors (in both **a** and **b**) on both their selective and alternative surface types. **Figure S4.** Swarming rates of evolved populations on alternative environment. Swarming rates of ancestors (gray) and each evolved population in its respective alternative environment, either soft agar (**a**) or hard agar (**b**). Evolved populations with a swarming rate that differed from their respective ancestors with *p* < 0.05 (paired Student *t*-test) are represented in red, whereas populations with *p* > 0.05 for difference from the ancestor are in black. Evolved populations are ordered left to right within each treatment set by increasing MyxoEE-3 population number. Swarming rates can be found in Additional file 1, Table S4. **c**. Average evolutionary change in swarming rates for each of the six treatments, expressed as a percentage increase relative to ancestor strains on their selective (as originally published in [47]) and alternative surfaces. Values shown are cross-population means of cross-replicate per-population means (*N* = 7, 8 or 12). *p < 0.05; **p < 0.01; ***p < 0.001, asterisks indicate significant effect of evolutionary treatment on swarming rate as calculated by one-sample *t*-tests for differences from 0. Error bars show 95% confidence intervals. **Figure S5.** Idiosyncratic and correlated evolution of alternative-surface swarming rates across MyxoEE-3 treatments. Plotted values represent means of evolved/ancestral swarming-rate ratios across at least three replicate experiments. Dashed lines indicate the standardized swarming rate of ancestors (*i.e.* 1.0). Absolute swarming rates in the selective environments were reported in [47]. Error bars show 95% confidence intervals. ‘HA’—hard agar; ‘SA’– soft agar. *p* values correspond to Spearman correlation tests. **Figure S6.** Alternative-surface swarming rate correlates positively with selective-surface swarming rate among single-motility-system populations without an outlier. Plotted values represent means of evolved/ancestral swarming-rate ratios across at least three replicate experiments, excluding the outlier population P56 included in Fig. 3a. Dashed line represents the linear model fit. *p* and rho values are from a Spearman correlation test excluding P56. **Figure S7.** Average mutation numbers in sequenced clones. Clones from dual-system vs. single system populations are compared (*t* test, *p* > 0.05). Horizontal lines indicate the average across clones within each category. One clone found to be a mutator (P29) [47] was excluded. **Table S1.** MyxoEE-3 treatments examined in this study. Minimum-generation estimates and other related information are presented in Table S1 of [47]. **Table S2.** Fitness of evolved populations on their alterative surface. Overall percentage of samples in which the kanamycin-marked ancestor was found at the edge of colonies that were initially composed as a 1:1 mix of the ancestor and an evolved population. Numbers in parentheses represent the number of population samples (out of five) collected from the swarm perimeter in which the ancestor was detected for each of the three replicate assays. Background shading reflects a heat map in which darkness correlates with the proportion of samples containing the ancestor. ‘HA’ and ‘SA’ indicate hard and soft agar, respectively. The corresponding data for competitions performed on each population’s MyxoEE-3 selective surface was reported in Table S2 of [47]. (The data for the control competitions between marked and unmarked ancestors on soft agar was omitted by oversight in [47] but is included here.) **Table S3.** Absolute swarming rates of each population on their selective and alternative surfaces. Data is expressed as mm/day. Green- and red-shaded cells correspond to significant increases and decreases in swarming rate compared to ancestor (paired *t*-student tests), respectively. ‘+’ and ‘–’ symbols for evolved populations indicate the direction of change relative to the proximate ancestor, irrespective of significance. Blue cells indicate populations with same-direction evolutionary-change estimates across surface types and tan cells indicate opposite-direction change estimates, irrespective of significance. **Table S4.** P values for differences in swarming on the alternative surface relative to the ancestor. Wilcoxon sign rank test for significant difference from 1. **Table S5.** Tests for correlations between evolutionary change in swarming rates on selective vs. alternative surfaces.

## Data Availability

The dataset supporting the results of this aricle is available in the Figshare repository, https://doi.org/10.6084/m9.figshare.12800909.v1 [87]

## Abbreviations

LPE: Latent-phenotype evolution
HA: Hard agar
SA: Soft agar
MyxoEE-3: Myxobacteria evolution experiment 3

## References

[1] Brakefield PM (2011). Evo-devo and accounting for Darwin's endless forms. Philos Trans R Soc Lond B Biol Sci.

[2] Carroll SB (2006). Endless forms most beautiful: the new science of Evo Devo and the making of the animal kingdom.

[3] Darwin CR (1859). On the origin of species by means of natural selection, or the preservation of favored races in the struggle for life.

[4] Gould SJ, Lewontin RC (1979). The spandrels of San Marco and the Panglossian paradigm: a critique of the adaptationist programme. Proc R Soc Lond B Biol Sci.

[5] Lynch M (2007). The evolution of genetic networks by non-adaptive processes. Nat Rev Genet.

[6] Paaby AB, Rockman MV (2013). The many faces of pleiotropy. Trends Genet.

[7] Hartfield M, Otto SP (2011). Recombination and hitchhiking of deleterious alleles. Evolution.

[8] Stephan W (2010). Genetic hitchhiking versus background selection: the controversy and its implications. Philos Trans R Soc Lond B Biol Sci.

[9] Griswold CK, Whitlock MC (2003). The genetics of adaptation: the roles of pleiotropy, stabilizing selection and drift in shaping the distribution of bidirectional fixed mutational effects. Genetics.

[10] Paaby AB, Rockman MV (2014). Cryptic genetic variation: evolution's hidden substrate. Nat Rev Genet.

[11] Buskirk SW, Peace RE, Lang GI (2017). Hitchhiking and epistasis give rise to cohort dynamics in adapting populations. Proc Natl Acad Sci USA.

[12] Elena SF, Lenski RE (2003). Evolution experiments with microorganisms: the dynamics and genetic bases of adaptation. Nat Rev Genet.

[13] Travisano M, Lenski RE (1996). Long-term experimental evolution in *Escherichia coli*. IV. Targets of selection and the specificity of adaptation. Genetics.

[14] Ebbert MA (1991). The interaction phenotype in the *Drosophila Willistoni*-Spiroplasma symbiosis. Evolution.

[15] Urban M (2020). PHI-base: the pathogen-host interactions database. Nucleic Acids Res.

[16] Coyne JA, Orr HA (2004). Speciation.

[17] Grant PR, Grant BR (2009). The secondary contact phase of allopatric speciation in Darwin's finches. Proc Natl Acad Sci USA.

[18] Podos J (2001). Correlated evolution of morphology and vocal signal structure in Darwin's finches. Nature.

[19] Rice WR, Hostert EE (1993). Laboratory experiments on speciation: what have we learned in 40 years?. Evolution.

[20] Blount ZD, Lenski RE, Losos JB (2018). Contingency and determinism in evolution: replaying life's tape. Science.

[21] Travisano M (1995). Experimental tests of the roles of adaptation, chance, and history in evolution. Science.

[22] Cooper VS, Lenski RE (2000). The population genetics of ecological specialization in evolving *Escherichia coli* populations. Nature.

[23] Solovieff N (2013). Pleiotropy in complex traits: challenges and strategies. Nat Rev Genet.

[24] Wang Z, Liao BY, Zhang J (2010). Genomic patterns of pleiotropy and the evolution of complexity. Proc Natl Acad Sci U S A.

[25] Le Nagard H, Chao L, Tenaillon O (2011). The emergence of complexity and restricted pleiotropy in adapting networks. BMC Evol Biol.

[26] Wagner GP (2008). Pleiotropic scaling of gene effects and the 'cost of complexity'. Nature.

[27] Welch JJ, Waxman D (2003). Modularity and the cost of complexity. Evolution.

[28] Bono LM (2017). The emergence of performance trade-offs during local adaptation: insights from experimental evolution. Mol Ecol.

[29] Futuyma DJ, Moreno G (1988). The Evolution of Ecological Specialization. Annu Rev Ecol Syst.

[30] Williams GC (1957). Pleiotropy, natural selection, and the evolution of senescence. Evolution.

[31] Phan K, Ferenci T (2013). A design-constraint trade-off underpins the diversity in ecologically important traits in species *Escherichia coli*. ISME J.

[32] Gifford DR, MacLean RC (2013). Evolutionary reversals of antibiotic resistance in experimental populations of *Pseudomonas aeruginosa*. Evolution.

[33] Torres-Barcelo C (2013). A trade-off between oxidative stress resistance and DNA repair plays a role in the evolution of elevated mutation rates in bacteria. Proc Biol Sci.

[34] Jasmin JN, Dillon MM, Zeyl C (2012). The yield of experimental yeast populations declines during selection. Proc Biol Sci.

[35] Spor A (2013). Phenotypic and genotypic convergences are influenced by historical contingency and environment in yeast. Evolution.

[36] Kubinak JL (2012). Experimental viral evolution to specific host MHC genotypes reveals fitness and virulence trade-offs in alternative MHC types. Proc Natl Acad Sci U S A.

[37] McNamara KB, Wedell N, Simmons LW (2013). Experimental evolution reveals trade-offs between mating and immunity. Biol Lett.

[38] Kreft JU, Bonhoeffer S (2005). The evolution of groups of cooperating bacteria and the growth rate versus yield trade-off. Microbiology.

[39] Novak M (2006). Experimental tests for an evolutionary trade-off between growth rate and yield in *E. coli*. Am Nat.

[40] Bennett AF, Lenski RE (2007). An experimental test of evolutionary trade-offs during temperature adaptation. Proc Natl Acad Sci U S A.

[41] Velicer GJ, Lenski RE (1999). Evolutionary trade-offs under conditions of resource abundance and scarcity: experiments with bacteria. Ecology.

[42] Wenger JW (2011). Hunger artists: yeast adapted to carbon limitation show trade-offs under carbon sufficiency. PLos Genet.

[43] Ackermann M (2007). Experimental evolution of aging in a bacterium. BMC Evol Biol.

[44] Taylor TB, Buckling A (2011). Selection experiments reveal trade-offs between swimming and twitching motilities in *Pseudomonas aeruginosa*. Evolution.

[45] van Ditmarsch D (2013). Convergent evolution of hyperswarming leads to impaired biofilm formation in pathogenic bacteria. Cell Rep.

[46] Nair RR, Fiegna F, Velicer GJ (2018). Indirect evolution of social fitness inequalities and facultative social exploitation. Proc R Biol Sci.

[47] Rendueles O, Velicer GJ (2017). Evolution by flight and fight: diverse mechanisms of adaptation by actively motile microbes. ISME J.

[48] Rendueles O (2015). Rapid and widespread de novo evolution of kin discrimination. Proc Natl Acad Sci U S A.

[49] Zee PC, Liu J, Velicer GJ (2017). Pervasive, yet idiosyncratic, epistatic pleiotropy during adaptation in a behaviourally complex microbe. J Evol Biol.

[50] Shimkets LJ (1999). Intercellular signaling during fruiting-body development of *Myxococcus xanthus*. Annu Rev Microbiol.

[51] Velicer GJ, Stredwick KL (2002). Experimental social evolution with *Myxococcus xanthus*. Antonie Van Leeuwenhoek.

[52] Velicer GJ, Vos M (2009). Sociobiology of the myxobacteria. Annu Rev Microbiol.

[53] Shi W, Zusman DR (1993). The two motility systems of *Myxococcus xanthus* show different selective advantages on various surfaces. Proc Natl Acad Sci U S A.

[54] Young IM, Crawford JW (2004). Interactions and self-organization in the soil-microbe complex. Science.

[55] Skerker JM, Berg HC (2001). Direct observation of extension and retraction of type IV pili. Proc Natl Acad Sci U S A.

[56] Wu SS, Kaiser D (1995). Genetic and functional evidence that Type IV pili are required for social gliding motility in *Myxococcus xanthus*. Mol Microbiol.

[57] Faure LM (2016). The mechanism of force transmission at bacterial focal adhesion complexes. Nature.

[58] Mignot T (2007). Evidence that focal adhesion complexes power bacterial gliding motility. Science.

[59] Hillesland KL, Velicer GJ (2005). Resource level affects relative performance of the two motility systems of *Myxococcus xanthus*. Microb Ecol.

[60] Spormann AM (1999). Gliding motility in bacteria: insights from studies of *Myxococcus xanthus*. Microbiol Mol Biol Rev.

[61] Velicer GJ, Yu YT (2003). Evolution of novel cooperative swarming in the bacterium *Myxococcus xanthus*. Nature.

[62] Youderian P (2003). Identification of genes required for adventurous gliding motility in *Myxococcus xanthus* with the transposable element mariner. Mol Microbiol.

[63] Youderian P, Hartzell PL (2006). Transposon insertions of magellan-4 that impair social gliding motility in *Myxococcus xanthus*. Genetics.

[64] Zee PC (2014). A shift from magnitude to sign epistasis during adaptive evolution of a bacterial social trait. Evolution.

[65] Rodriguez AM, Spormann AM (1999). Genetic and molecular analysis of cglB, a gene essential for single-cell gliding in *Myxococcus xanthus*. J Bacteriol.

[66] Wiser MJ, Ribeck N, Lenski RE (2013). Long-term dynamics of adaptation in asexual populations. Science.

[67] Niklas KJ, Newman SA (2019). The many roads to (and from) multicellularity. J Exp Bot..

[68] Ratcliff WC, Travisano M (2014). Experimental evolution of multicellular complexity in *Saccharomyces cerevisiae*. Bioscience.

[69] Vos M, Velicer GJ (2008). Natural variation of gliding motility in a centimetre-scale population of *Myxococcus xanthus*. FEMS Microbiol Ecol.

[70] Blount ZD, Borland CZ, Lenski RE (2008). Historical contingency and the evolution of a key innovation in an experimental population of *Escherichia coli*. Proc Natl Acad Sci USA.

[71] Gleizer S (2019). Conversion of *Escherichia coli* to generate all biomass carbon from CO_2_. Cell.

[72] Smith J, Van Dyken JD, Velicer GJ (2014). Nonadaptive processes can create the appearance of facultative cheating in microbes. Evolution.

[73] Velicer GJ, Kroos L, Lenski RE (2000). Developmental cheating in the social bacterium *Myxococcus xanthus*. Nature.

[74] Vasi F, Travisano M, Lenski RE (1994). Long-term experimental evolution in *Escherichia coli*. 2. Changes in life-history traits during adaptation to a seasonal environment. Am Nat.

[75] Clune J, Mouret JB, Lipson H (2013). The evolutionary origins of modularity. Proc Biol Sci.

[76] Wagner GP, Zhang J (2011). The pleiotropic structure of the genotype–phenotype map: the evolvability of complex organisms. Nat Rev Genet.

[77] Cooper TF, Lenski RE (2010). Experimental evolution with *E. coli* in diverse resource environments. I. Fluctuating environments promote divergence of replicate populations. BMC Evol Biol..

[78] Dos Santos M, Ghoul M, West SA (2018). Pleiotropy, cooperation, and the social evolution of genetic architecture. PLoS Biol.

[79] Foster KR (2004). Pleiotropy as a mechanism to stabilize cooperation. Nature.

[80] Velicer GJ, Kroos L, Lenski RE (1998). Loss of social behaviors by *Myxococcus xanthus* during evolution in an unstructured habitat. Proc Natl Acad Sci U S A.

[81] Fiegna F, Velicer GJ (2003). Competitive fates of bacterial social parasites: persistence and self-induced extinction of *Myxococcus xanthus* cheaters. Proc R Soc Lond Biol Sci.

[82] Fiegna F (2006). Evolution of an obligate social cheater to a superior cooperator. Nature.

[83] Hillesland KL, Velicer GJ, Lenski RE (2009). Experimental evolution of a microbial predator's ability to find prey. Proc Biol Sci.

[84] Manhes P, Velicer GJ (2011). Experimental evolution of selfish policing in social bacteria. Proc Natl Acad Sci U S A.

[85] Nair RR (2019). Bacterial predator-prey coevolution accelerates genome evolution and selects on virulence-associated prey defences. Nat Commun.

[86] Velicer GJ (2006). Comprehensive mutation identification in an evolved bacterial cooperator and its cheating ancestor. Proc Natl Acad Sci U S A.

[87] Rendueles O. 2020. Raw_data_Rendueles&Velicer.xlsx.figshare.Dataset. 10.6084/m9.figshare.12800909.v1.

