# Supplementary material for: Hidden paths to endless forms most wonderful: Complexity of bacterial motility shapes diversification of latent phenotypes
Source: BMC Evol Biol. 2020 Nov 4;20:145. doi: 10.1186/s12862-020-01707-3 (PMC7641858; doi:10.1186/s12862-020-01707-3)
Supplement: Supplementary file 1 — Additional file 1: Figure S1. Alternative-surface fitness patterns across treatments: per-population means. The leading edges of swarming colonies initially mixed as 1:1 evolved:marked-ancestor were sampled for the presence/absence of the marked ancestor (five samples per colony). The mean ancestor-presence proportions for each evolved population across three replicate assays are shown (data points). Gray bars correspond to the within-treatment average of the per-population means. Data for the selective-surface assays was originally published in [47]. * p < 0.05, ***p < 0.001; Student’s t-test for proportion differences between selective vs. alternative surfaces. Error bars show 95% confidence intervals. Figure S2. Colony-phenotype diversification of A−S+ populations on both surface types. Colony phenotypes of (a) eight populations evolved on hard agar (HA), (b) seven populations evolved on soft agar (SA) and their ancestors (in both a and b) on both their selective and alternative surface types. Figure S3. Colony-phenotype diversification of A+S- populations on both surface types. Colony phenotypes of (a) eight populations evolved on hard agar (HA), (b) eight populations evolved on soft agar (SA) and their ancestors (in both a and b) on both their selective and alternative surface types. Figure S4. Swarming rates of evolved populations on alternative environment. Swarming rates of ancestors (gray) and each evolved population in its respective alternative environment, either soft agar (a) or hard agar (b). Evolved populations with a swarming rate that differed from their respective ancestors with p < 0.05 (paired Student t-test) are represented in red, whereas populations with p > 0.05 for difference from the ancestor are in black. Evolved populations are ordered left to right within each treatment set by increasing MyxoEE-3 population number. Swarming rates can be found in Additional file 1, Table S4. c. Average evolutionary change in swarming rates for each of the [file 12862_2020_1707_MOESM1_ESM.docx]

**SUPPLEMENTARY TABLES**

**Supplementary Table S1. MyxoEE-3 treatments examined in this study.** Minimum-generation estimates and other related information are presented in Table S1 of [47]

| MyxoEE-3  selective surface | Motility  genotype | Ancestor  strain | Formal  genotype | Motility-system contributions to swarming | Antibiotic  phenotype | Replicate populations  per ancestor | Population labels |
| --- | --- | --- | --- | --- | --- | --- | --- |
| Hard agar | A+S+ | GJV1 | *cglB*^+^ *pilA*^+^ | Both A and S motility | Rif^S^ | 6 | P1, P3, P5  P7, P9, P11 |
|  | A+S+ | GJV2 | *cglB*^+^ *pilA*^+^ | Both A and S motility | Rif^R^ | 6 | P2, P4, P6,  P8, P10, P12 |
|  | A-S+ | GJV3 | Δ*cglB pilA*^+^ | Only S motility | Rif^S^ | 4 | P13, P15, P17, P19 |
|  | A-S+ | GJV5 | Δ*cglB*  *pilA*^+^ | Only S motility | Rif^R^ | 4 | P14 ^b^, P16, P18, P20 |
|  | A+S- | GJV4 | *cglB*^+^ Δ*pilA* | Only A motility | Rif^S^ | 4 | P21, P23, P25, P27 |
|  | A+S- | GJV6 | *cglB*^+^ Δ*pilA* | Only A motility | Rif^R^ | 4 | P22, P24, P26, P28 |
| Soft agar | A+S+ | GJV1 | *cglB*^+^ *pilA*^+^ | Primarily S motility | Rif^S^ | 6 | P29^a^, P31, P33  P35, P37, P39 |
|  | A+S+ | GJV2 | *cglB*^+^ *pilA*^+^ | Primarily S motility | Rif^R^ | 6 | P30, P32, P34  P36, P38, P40 |
|  | A-S+ | GJV3 | Δ*cglB pilA*^+^ | Only S motility | Rif^S^ | 3 | P41, P43, P45 |
|  | A-S+ | GJV5 | Δ*cglB*  *pilA*^+^ | Only S motility | Rif^R^ | 4 | P42, P44, P46, P48 |
|  | A+S- | GJV4 | *cglB*^+^ Δ*pilA* | Only A motility | Rif^S^ | 4 | P49, P51, P53, P55 |
|  | A+S- | GJV6 | *cglB*^+^ Δ*pilA* | Only A motility | Rif^R^ | 4 | P50, P52, P54, P56 |

‘Rif’ = rifampicin, ‘S’ = sensitive, ‘R’ = resistant

**^a^** P29 is a hypermutator, it has been removed from genetic analyses but not phenotypic analyses

^b^ P14 was examined after 36 cycles of MyxoEE-3. All other populations were examined after 40 cycles.

**Table S2. Fitness of evolved populations on their alterative surface.** Overall percentage of samples in which the kanamycin-marked ancestor was found at the edge of colonies that were initially composed as a 1:1 mix of the ancestor and an evolved population. Numbers in parentheses represent the number of population samples (out of five) collected from the swarm perimeter in which the ancestor was detected for each of the three replicate assays. Background shading reflects a heat map in which darkness correlates with the proportion of samples containing the ancestor. ‘HA’ and ‘SA’ indicate hard and soft agar, respectively. The corresponding data for competitions performed on each population’s MyxoEE-3 selective surface was reported in Table S2 of [47]. (The data for the control competitions between marked and unmarked ancestors on soft agar was omitted by oversight in [47] but is included here.)

| MyxoEE-3 treatment | Population or genotype | Marked-ancestor presence % | Population or genotype | Marked-ancestor presence % | Alternative  (assay) surface |
| --- | --- | --- | --- | --- | --- |
| Ancestors | A+S+ | 100 | rA+S+ | 100 | CTT HA |
|  | A-S+ | 100 | rA-S+ | 100 |  |
|  | A+S- | 100 | rA+S- | 100 |  |
| Ancestor | A+S+ | 93 (5,5,4) | rA+S+ | 100 | CTT SA |
|  | A-S+ | 93 (5,5,4) | rA-S+ | 100 |  |
|  | A+S- | 100 | rA+S- | 100 |  |
| A+S+  CTT HA | P1 | 0 | P2 | 13 (0,2,2) | CTT SA |
|  | P3 | 0 | P4 | 33 (0,2,3) |  |
|  | P5 | 0 | P6 | 27 (0,0,4) |  |
|  | P7 | 13 (1,0,1) | P8 | 27 (0,1,3) |  |
|  | P9 | 33 (0,0,5) | P10 | 33 (0,0,5) |  |
|  | P11 | 27 (0,0,4) | P12 | 7 (1,0,0) |  |
| A-S+  CTT HA | P13 | 0 | P14 | 0 | CTT SA |
|  | P15 | 0 | P16 | 0 |  |
|  | P17 | 20 (0,0,3) | P18 | 0 |  |
|  | P19 | 0 | P20 | 0 |  |
| A+S-  CTT HA | P21 | 67 (0,5,5) | P22 | 100 | CTT SA |
|  | P23 | 100 | P24 | 53 (5,3,0) |  |
|  | P25 | 93 (4,5,5) | P26 | 100 |  |
|  | P27 | 100 | P28 | 100 |  |
| A+S+  CTT SA | P29 | 0 | P30 | 0 | CTT HA |
|  | P31 | 0 | P32 | 27 (3,1,0) |  |
|  | P33 | 0 | P34 | 0 |  |
|  | P35 | 100 | P36 | 60 (4,0,5) |  |
|  | P37 | 0 | P38 | 0 |  |
|  | P39 | 73 (5,2,4) | P40 | 0 |  |
| A-S+  CTT SA | P41 | 67 (5,0,5) | P42 | 0 | CTT HA |
|  | P43 | 0 | P44 | 73 (4,2,5) |  |
|  | P45 | 7 (0,0,1) | P46 | 13 (0,0,2) |  |
|  |  |  | P48 | 0 |  |
| A+S-  CTT SA | P49 | 60 (5,0,4) | P50 | 53 (3,1,4) | CTT HA |
|  | P51 | 0 | P52 | 0 |  |
|  | P53 | 60 (0,5,4) | P54 | 100 |  |
|  | P55 | 7 (1,0,0) | P56 | 67 (5,3,2) |  |

**Table S3. Absolute swarming rates of each population on their selective and alternative surfaces.** Data is expressed as mm/day. Green and red cells correspond to significant increases and decreases in swarming rate compared to ancestor (paired t-student tests), respectively. ‘+’ and ‘–’ symbols for evolved populations indicate the direction of change relative to the proximate ancestor, irrespective of significance. Blue cells indicate populations with same-direction evolutionary-change estimates across surface types and tan cells indicate opposite-direction change estimates, irrespective of significance.

|  |  |  | **Selective surface** | **Alternative surface** |  |  |  |  | **Selective surface** | **Alternative surface** |  |
| --- | --- | --- | --- | --- | --- | --- | --- | --- | --- | --- | --- |
|  | **Ancestors** | A+S+ | 3.03 | 2.39 |  |  | **Ancestors** | A+S+ | 2.39 | 3.03 |  |
|  |  | rA+S+ | 3.25 | 2.33 |  |  |  | rA+S+ | 2.33 | 3.25 |  |
|  |  | A-S+ | 1.65 | 2.24 |  |  |  | A-S+ | 2.24 | 1.65 |  |
|  |  | rA-S+ | 1.69 | 2.08 |  |  |  | rA-S+ | 2.08 | 1.69 |  |
|  |  | A+S- | 1.03 | 0.54 |  |  |  | A+S- | 0.54 | 1.03 |  |
|  |  | rA+S- | 1.18 | 0.42 |  |  |  | rA+S- | 0.42 | 1.18 |  |
| **Evolved on hard agar** | **A+S+** | P1 | 3.26 + | 1.83 – |  | **Evolved on soft agar** | **A+S+** | P29 | **3.44 +** | 3.65 + |  |
|  |  | P2 | 3.43 + | **3.47 +** |  |  |  | P30 | **4.54 +** | 3.33 + |  |
|  |  | P3 | 3.06 + | 2.39 = |  |  |  | P31 | 2.11 – | 3.08 + |  |
|  |  | P4 | **3.69 +** | **4.56 +** |  |  |  | P32 | **3.97 +** | 3.17 + |  |
|  |  | P5 | **3.59 +** | 1.36 – |  |  |  | P33 | 3.03 + | **3.50 +** |  |
|  |  | P6 | 3.10 – | 2.68 + |  |  |  | P34 | **3.14 +** | 3.03 – |  |
|  |  | P7 | 3.08 + | 2.14 – |  |  |  | P35 | 2.26 – | 2.79 – |  |
|  |  | P8 | **3.68 +** | **2.83 +** |  |  |  | P36 | 2.07 – | **1.96 –** |  |
|  |  | P9 | 3.21 + | 1.58 – |  |  |  | P37 | 3.07 + | 2.76 – |  |
|  |  | P10 | 3.19 – | **2.81 +** |  |  |  | P38 | **2.63 +** | **3.67 +** |  |
|  |  | P11 | **2.79 –** | 2.74 + |  |  |  | P39 | 2.36 – | 2.62 – |  |
|  |  | P12 | 3.31 + | 3.04 + |  |  |  | P40 | **1.81 –** | **4.04 +** |  |
|  | **A-S+** | P13 | **2.50 +** | **3.93 +** |  |  | **A-S+** | P41 | **1.38 –** | **1.10 –** |  |
|  |  | P14 | **2.67 +** | 2.06 – |  |  |  | P42 | **4.50 +** | **2.54 +** |  |
|  |  | P15 | **2.58 +** | 2.06 – |  |  |  | P43 | **3.81 +** | **3.77 +** |  |
|  |  | P16 | **2.74 +** | 2.78 + |  |  |  | P44 | 1.11 – | 1.72 + |  |
|  |  | P17 | **2.24 +** | 2.24 = |  |  |  | P45 | **3.39 +** | **2.50 +** |  |
|  |  | P18 | **2.21 +** | **4.28 +** |  |  |  | P46 | 1.78 – | **1.18 –** |  |
|  |  | P19 | **2.50 +** | **3.17 +** |  |  |  |  |  |  |  |
|  |  | P20 | 1.97 + | 1.72 – |  |  |  | P48 | 2.18 + | **1.88 +** |  |
|  | **A+S-** | P21 | **1.71 +** | 0.42 – |  |  | **A+S-** | P49 | 0.57 + | 0.79 – |  |
|  |  | P22 | **1.49 +** | 0.39 – |  |  |  | P50 | **1.03 +** | **1.79 +** |  |
|  |  | P23 | **1.65 +** | 0.51– |  |  |  | P51 | **0.94 +** | **2.29 +** |  |
|  |  | P24 | **2.27 +** | **1.04 +** |  |  |  | P52 | 0.42 = | 1.43 + |  |
|  |  | P25 | **1.56 +** | 0.33 – |  |  |  | P53 | 0.53 – | **1.40 +** |  |
|  |  | P26 | **2.63 +** | 0.67 + |  |  |  | P54 | 0.42 = | 0.92 – |  |
|  |  | P27 | **1.76 +** | 0.39 – |  |  |  | P55 | **1.39 +** | **2.32 +** |  |
|  |  | P28 | **1.38 +** | 0.32 – |  |  |  | P56 | **2.78 +** | **2.07 +** |  |

‘+’ and ‘–’ for evolved populations indicate the direction of change relative to the proximate ancestor.

**Table S4. P values for differences in swarming on the alternative surface relative to the ancestor.** Wilcoxon sign rank test for significant difference from 1.

|  |  | V | P value |
| --- | --- | --- | --- |
| Hard agar | A+S+ | 52 | 0.33 |
|  | A-S+ | 28 | 0.19 |
|  | A+S- | 15 | 0.74 |
| Soft agar | A+S+ | 40 | 0.97 |
|  | A-S+ | 21 | 0.3 |
|  | A+S- | 31 | 0.08 |

**Table S5. Tests for correlations between evolutionary change in swarming rates on selective vs. alternative surfaces**

| MyxoEE-3 selective  surface | Ancestral  motility genotype | *N* | Spearman’s  *ρ* | *P* |
| --- | --- | --- | --- | --- |
|  | A+S+ | 24 | -0.1 | 0.65 |
|  | mutants combined | 31 | 0.58 | **<0.001** |
| Combined | A-S+ | 15 | 0.55 | **0.04** |
|  | A+S- | 16 | 0.56 | **0.02** |
| Hard agar | combined | 28 | 0.07 | 0.97 |
| Soft agar |  | 27 | 0.64 | **<0.001** |
| Hard agar | A+S+ | 12 | -0.11 | 0.73 |
|  | mutants combined | 16 | 0.13 | 0.62 |
|  | A-S+ | 8 | 0.04 | 0.93 |
|  | A+S- | 8 | 0.79 | 0.30 |
| Soft agar | A+S+ | 12 | 0.13 | 0.68 |
|  | mutants combined | 15 | 0.77 | **0.001** |
|  | A-S+ | 7 | 0.93 | **0.04** |
|  | A+S- | 8 | 0.62 | 0.12 |

**SUPPLEMENTARY FIGURES**

**Figure S1. Alternative-surface fitness patterns across treatments: per-population means.**The leading edges of swarming colonies initially mixed as 1:1 evolved:marked-ancestor were sampled for the presence/absence of the marked ancestor (five samples per colony). The mean ancestor-presence proportions for each evolved population across three replicate assays are shown (data points). Gray bars correspond to the within-treatment average of the per-population means.  Data for the selective-surface assays was originally published in [47]**. *** *p* < 0.05, ****p* < 0.001; Student’s *t-*test for proportion differences between selective vs alternative surfaces. Error bars show 95% confidence intervals.

**
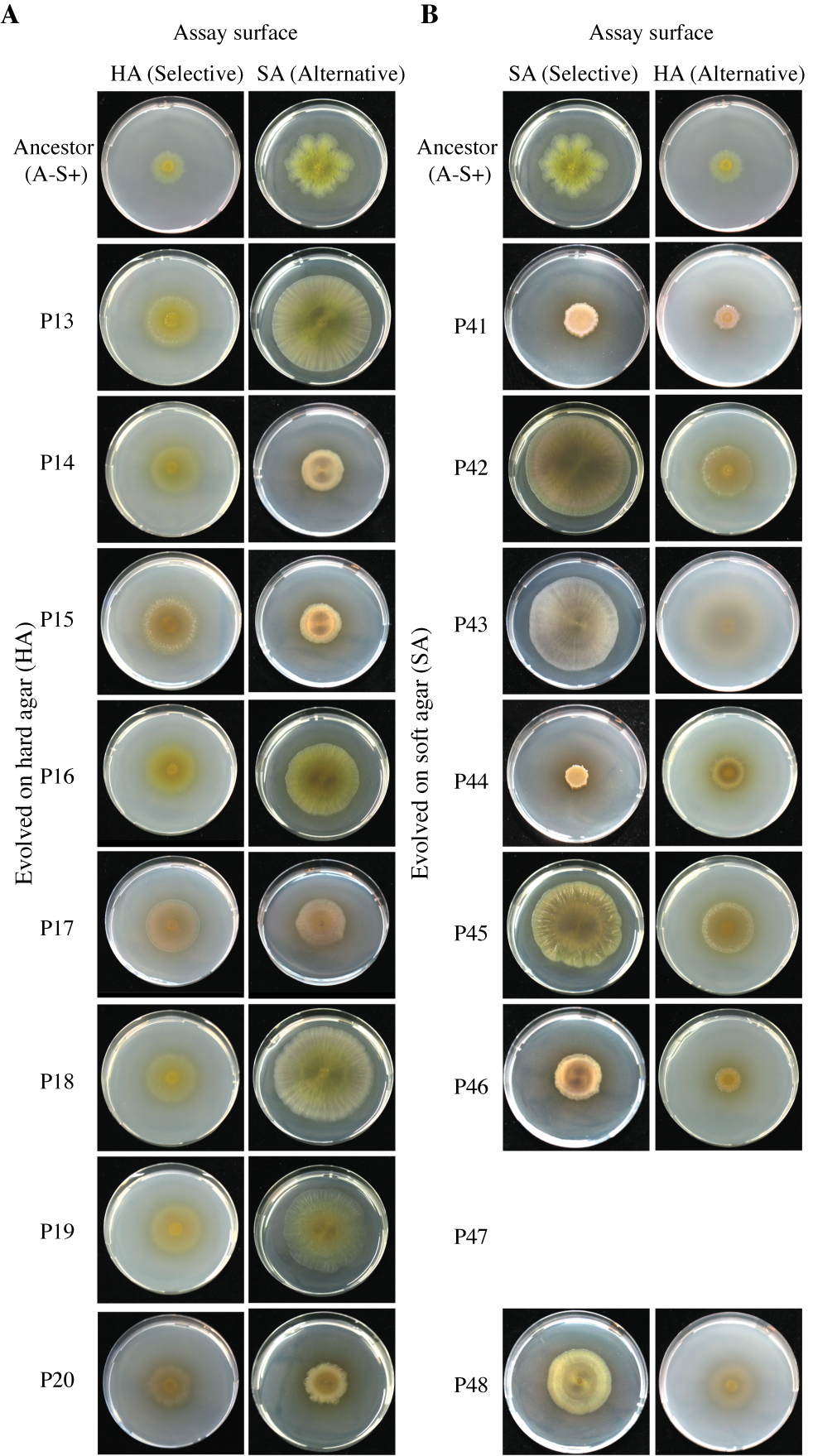
**

**Figure S2. Colony-phenotype diversification of A-S+ populations on both surface types.** Colony phenotypes of **(A)** eight populations evolved on hard agar (HA), **(B)** seven populations evolved on soft agar (SA) and their ancestors (in both **A** and **B**) on both their selective and alternative surface types.

**
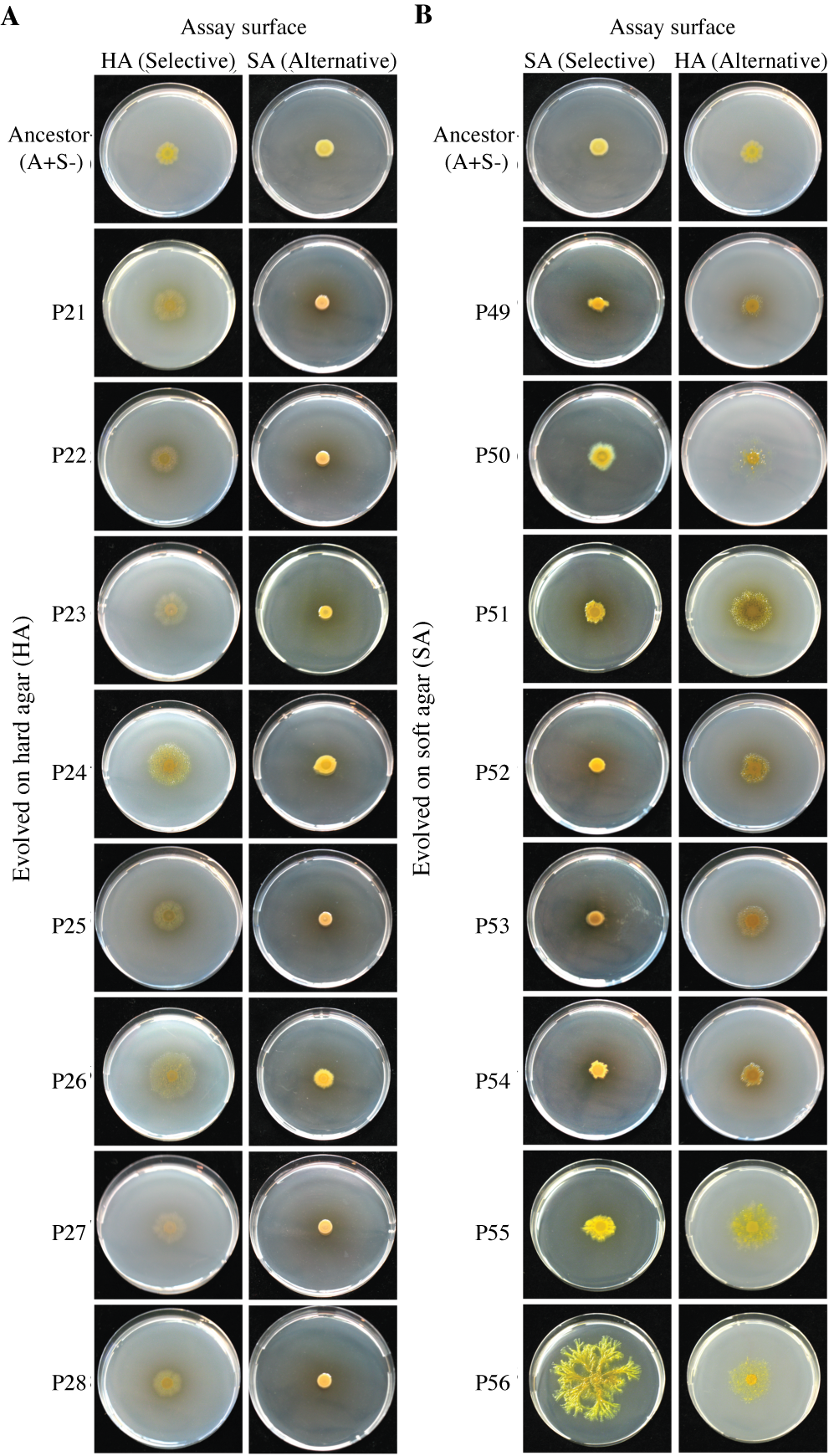
**

**Figure S3. Colony-phenotype diversification of A+S- populations on both surface types.** Colony phenotypes of **(A)** eight populations evolved on hard agar (HA), **(B)** eight populations evolved on soft agar (SA) and their ancestors (in both **A** and **B**) on both their selective and alternative surface types.

**
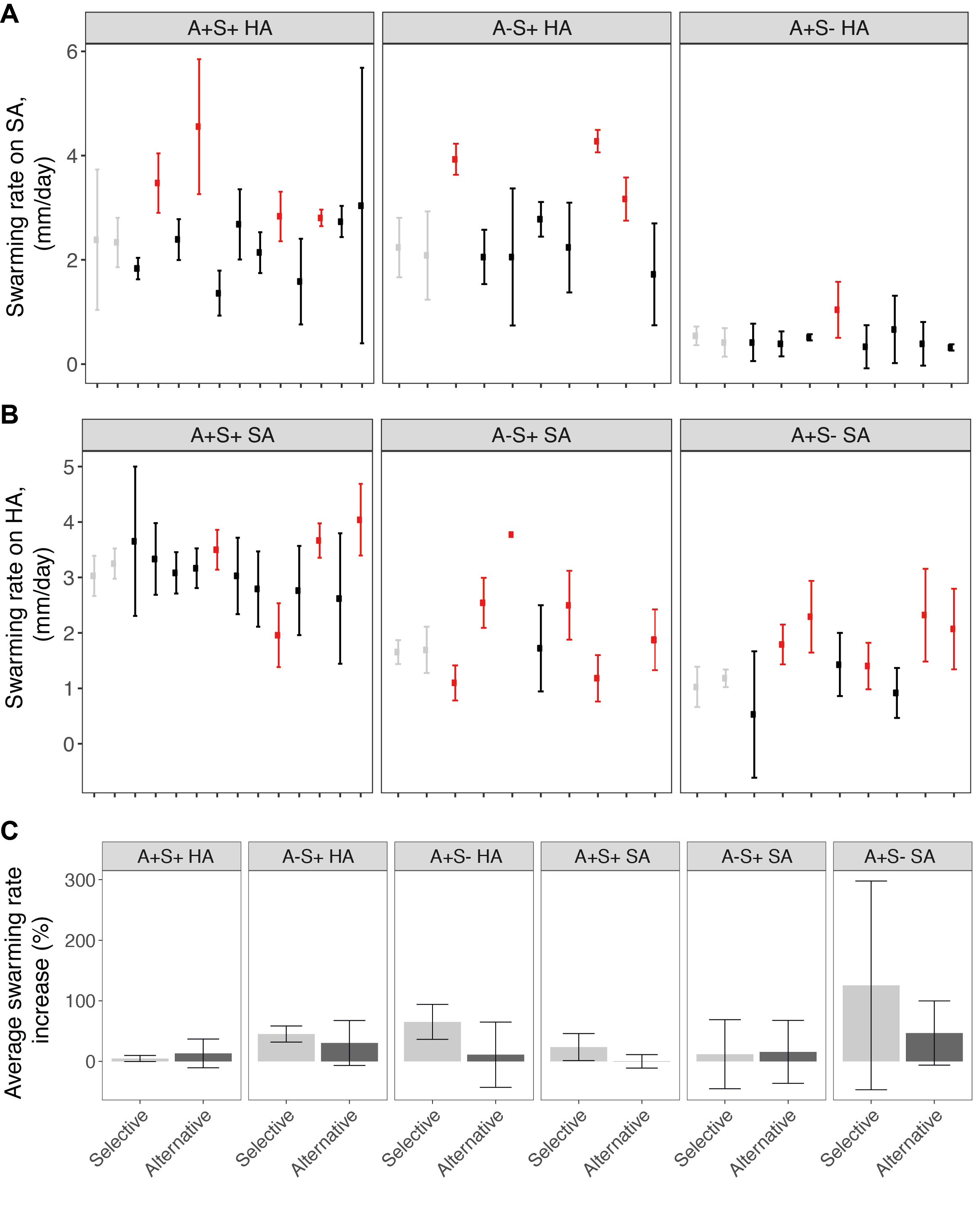
**

**Figure S4. Swarming rates of evolved populations on alternative environment.** Swarming rates of ancestors (gray) and each evolved population in its respective alternative environment, either soft agar (**A**) or hard agar **(B)**. Evolved populations with a swarming rate that differed from their respective ancestors with *p* < 0.05 (paired Student t-test) are represented in red, whereas populations with *p >* 0.05 for difference from the ancestor are in black. Evolved populations are ordered left to right within each treatment set by increasing MyxoEE-3 population number. Swarming rates can be found in Supplementary Table S4. **C**. Average evolutionary change in swarming rates for each of the six treatments, expressed as a percentage increase relative to ancestor strains on their selective (as originally published in [47]) and alternative surfaces. Values shown are cross-population means of cross-replicate per-population means (*N =* 7, 8 or 12). **p* < 0.05; ***p* < 0.01; ****p* < 0.001, asterisks indicate significant effect of evolutionary treatment on swarming rate as calculated by one-sample *t*-tests for differences from 0. Error bars show 95% confidence intervals.

**Fig. S5. Idiosyncratic and correlated evolution of alternative-surface swarming rates across MyxoEE-3 treatments.** Plotted values represent means of evolved/ancestral swarming-rate ratios across at least three replicate experiments. Dashed lines indicate the standardized swarming rate of ancestors (*i.e.* 1.0). Absolute swarming rates in the selective environments were reported in [47]. Error bars show 95% confidence intervals. ‘HA’ – hard agar; ‘SA’– soft agar. *p* values correspond to Spearman correlation tests.

**Figure S6. Alternative-surface swarming rate correlates positively with selective-surface swarming rate among single-motility-system populations.** Plotted values represent means of evolved/ancestral swarming-rate ratios across at least three replicate experiments. Dashed line represents the linear model fit. *p* and rho values correspond to Spearman correlation tests, excluding population P56, an outlier in the dataset.

**Figure S7. Average mutation numbers in sequenced clones.** Clones from dual-system vs single system populations are compared (*t* test, *p* > 0.05). Horizontal lines indicate the average across clones within each category. One clone found to be a mutator (P29) [47] was excluded.
